# Predictive coding explains asymmetric connectivity in the brain: A neural network study

**DOI:** 10.1371/journal.pcbi.1014435

**Published:** 2026-07-06

**Authors:** Romesa Khan, Hongsheng Zhong, Shuvam Das, Jack Cai, Matthias Niemeier

**Affiliations:** 1 Department of Psychology, University of Toronto Scarborough, Toronto, Ontario, Canada; 2 Department of Computer Science, University of Toronto, Toronto, Ontario, Canada; 3 Department of Electrical and Computer Engineering, University of Toronto, Toronto, Ontario, Canada; 4 Centre for Vision Research, York University, Toronto, Ontario, Canada; 5 Vision: Science to Applications, York University, Toronto, Ontario, Canada; Basque Center on Cognition Brain and Language, SPAIN

## Abstract

Seminal frameworks of predictive coding propose a hierarchy of generative modules, each attempting to infer the neural representation of the module one level below; the predictions are carried by top-down feedback projections, while the predictive error is propagated by reciprocal forward pathways. Such symmetric feedback connections support visual processing of noisy stimuli in computational models. However, neurophysiological studies have yielded evidence of asymmetric cortical feedback connections. We investigated the contribution of neural feedback in visual processing for computing grasp parameters, by utilizing convolutional neural network models that had been augmented with predictive feedback and were trained to compute grasp positions for real-world objects. After establishing an ameliorative effect of symmetric feedback on grasp detection performance when evaluated on noisy stimuli, we characterized the performance effects of asymmetric feedback, similar to that observed in the cortex. Specifically, we tested model variants extended with *short*-, *medium*-, *long*- and *longer*-range feedback connections (i) originating at the same source layer or (ii) terminating at the same target layer. We found that the performance-enhancing effect of predictive coding under adverse conditions was optimal for *medium*-range asymmetric feedback. Moreover, this effect was most prominent when *medium*-range feedback originated at a level of representational abstraction that was proximal to the input layer, in contrast to more distal layers. To conclude, our simulations show that introducing biologically realistic asymmetric predictive feedback improves model robustness to noisy visual stimuli in a neural network model optimized for grasp detection.

## Author summary

Everyday vision is noisy: objects can be blurred, partly hidden, or poorly illuminated. Predictive coding is a commonly held idea in neuroscience, which proposes that higher brain areas send feedback to lower areas to “clean up” sensory information. But how far should these feedback signals travel, and from which level should they originate? We studied this question with deep neural networks that plan how to grasp objects from noisy images. We added feedback connections to the networks and varied two factors independently: the path length of feedback and the level of abstraction (information richness) of its source. Across varying degrees of image noise, medium-range feedback from relatively early stages of the network produced the most reliable improvements. These results suggest that intermediate visual stages in the brain may be well placed to send predictive signals that stabilize visual representations for computing grasp parameters. Beyond informing neuroscience, our findings provide design principles for making machine perception and future robotic grasping systems more robust to noisy inputs.

## Introduction

Descending neural pathways transmit neural signals from higher cortical areas back to earlier processing stages, enabling top-down modulation of lower-level neural circuits. Such feedback has been recognized as central for perceptual and cognitive processes, enabling the brain to implement learning mechanisms [Roelfsema & Holtzman, 2018], attentional modulation [1,2] and refinement of sensory representations based on context, expectations, and prior experience [3,4,5]. Crucially, feedback is believed to be instrumental for conveying predictions.

That is, the brain has been widely conceptualized as a “prediction machine” that relies on internal generative models to actively construct explanations for the causes of noisy sensory inputs [6]. Specifically, it is held that predictive feedback signals are conveyed via cortico-cortical top-down projections that are then compared to bottom-up signals such that the deviations from these signals, in the form of predictions errors, are carried forward. An influential framework of such predictive coding proposes a hierarchy of generative modules, each attempting to infer the neural representation of the module below, with topographically reciprocal feedforward connections [7,8,9,10]. This idea maps well on previous anatomical data: Felleman and Van Essen [11] proposed a hierarchical model of the visual cortex where adjacent areas often maintain both feedforward and feedback pathways, implying a measure of reciprocity in the network [12,13]. Indeed, tracing studies in macaques reveal that reciprocal links between closely related visual areas significantly account for interareal communication [12]. Such symmetric feedback connections are particularly important for object recognition under noisy or ambiguous viewing conditions. Hupé et al. [14] have shown that area V2 modulates V1 responses, enhancing the detection of figures embedded in cluttered or noisy images. Furthermore, recurrent interactions between V1 and higher visual areas, including V2, are crucial for figure-ground segregation [15]. Similarly, in computer simulations, symmetric feedback connections support object discrimination [16], particularly when processing noisy stimuli [17,18].

However, there is growing evidence of asymmetric, non-reciprocated cortical feedback connections from quantitative mapping studies [19,20]. Long-range descending pathways cascade over multiple cortical areas [21,22], and an advantage of long-latency over short-latency visuomotor feedback has been observed during cortical reward processing [23]. Furthermore, experiments studying visuospatial attention in primates indicate a functional role of *medium*-range predictive feedback, extending from area V4 to area V1, in encoding accuracy of input stimuli [24], suggesting a potential benefit of *medium*-range feedback in signal processing under noise. This is similar to the previously suggested role of *medium*-range feedback in disambiguating signal from noise during global contour integration [25].

To investigate the contribution of asymmetrical neural feedback, here we simulated grasp prediction utilizing convolutional neural networks (CNNs) as a modelling framework. Hierarchical CNN architectures have been commonly used for object recognition tasks [26] and are posited as suitable models of vision in the primate brain [27,28]. Crucially, rather than solely relying on the feedforward flow of information in the canonical architecture of CNNs, we augmented them with symmetric, or asymmetric generative feedback loops that carried advanced representations to earlier layers of the networks, mimicking various aspects of feedback in the biological brain. The aim of the study was to understand (a) if an ameliorative effect of feedback can be isolated during visual processing for computing grasp parameters when the incoming signal is corrupted, and (b) to explore the layer-dependence of such predictive feedback in our model, thus shedding light on the relative functional contribution of cortico-cortical connectivity of asymmetric feedback originating at varying levels of representational abstraction.

## Results

We investigated the contribution of feedback to visual processing for computing grasp parameters, using a neural network model that was optimized to detect grasp points on objects.

### Feedback improves robustness of grasp detection to noise

**Fig 1:**
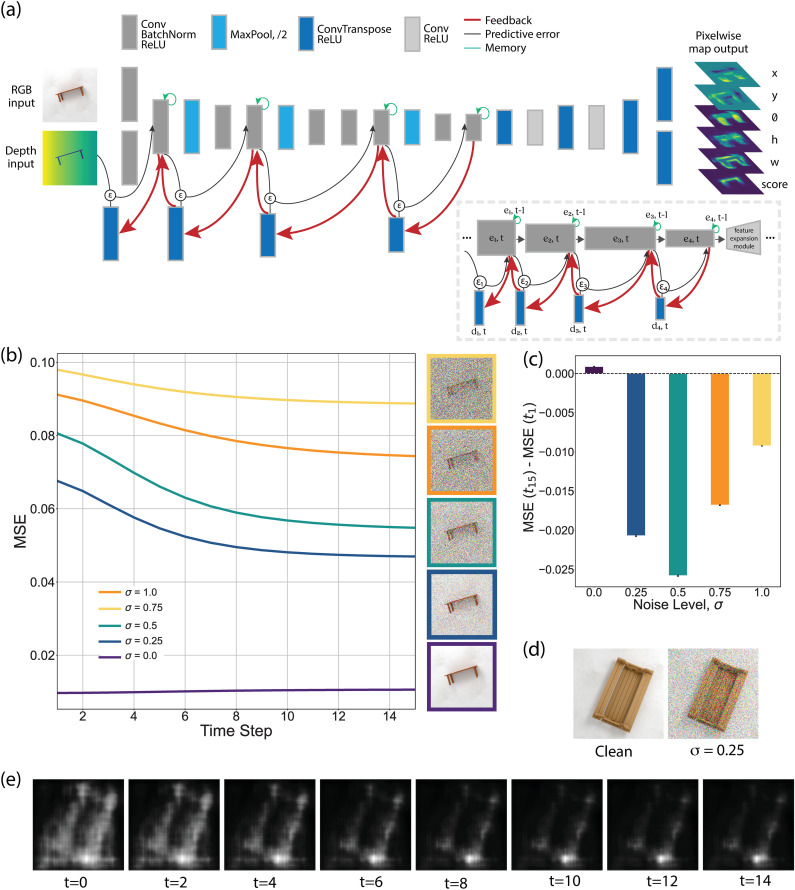
Predictive coding dynamics improve model performance for noisy stimuli. **(a)** Architecture of the grasping neural network, derived from the short feedforward backbone. Red arrows denote top-down feedback connections. Black arrows represent the error-correction process, whereby the predictive error (ϵ) drives the higher layer (feedback source) representation to better match the early layer representation (feedback target). Green arrows denote recurrent memory processes, for conserving the layer representation over consecutive predictive cycles. Conv: convolutional layer; BatchNorm: batch normalization layer; ReLU: rectifying linear unit; MaxPool: pooling layer; ConvTranspose: transpose convolutional layer. The network received a 3-channel RGB image and a 1-channel depth image of the input object [29]. The network output consisted of a 6-channel array, each channel representing a pixelwise map with the same dimensions as the input images and corresponding to one of the 5 grasp parameters: grasp centre coordinates (x, y); orientation (θ); grasp opening (h); gripper size (w) and the grasp quality (confidence) score. Inset illustrates how the model layers are organized into sequential PCoders. **(b)** Model performance during the inference phase, across 15 predictive coding timesteps, measured as the average mean squared error at each timestep, when presented with the test dataset that had been injected with varying levels of additive Gaussian noise. The right panel shows a representative sample object, corrupted with the different noise levels. **(c)** The average difference in test performance of the model between the initial (t = 1) and final (t = 15) timestep, for each noise level. Black bars denote standard errors of mean. **(d)** Left panel: an RGB image of a sample object from the test dataset [29]. Right panel: the RGB image with Gaussian noise (σ=0.25). **(e)** The grasp quality score maps of the sample object, at even timesteps. RGB and depth images in parts **(a)**, **(b)** and **(d)** were reused with permission from the authors of the Jacquard dataset.

We evaluated the effect of predictive feedback on the performance of the trained model, when presented with adversarial images that were injected with varying levels of Gaussian noise (σ={0, 0.25, 0.5, 0.75, 1.0}) (Experiment 1 in Methods). In effect, the performance of a feedback model at the first timestep (t=1) of the iterative predictive coding dynamics is identical to the performance of the corresponding feedforward backbone. This backbone performance was compared to the performance of the feedback model after several recurrent timesteps (t=15) after which performance no longer changed. We observed an improvement in the grasp detection accuracy of the model after predictive coding iterations, across all levels of Gaussian noise (Fig 1B; n.b., there was a small decline in performance for zero noise). The average difference in model performance between the first (t=0) and the final (t=15) timestep was significant across noise levels, F(4, 22267)=220036.00,p<.001 (Fig 1C). Fig 1E depicts progressive noise removal in the model output (represented by the grasp quality score map) across successive predictive coding timesteps, for a sample object from the test dataset (Fig 1D).

### Performance-related effects of feedback are layer-dependent

Next, we tested whether the performance-enhancing effects of feedback were contingent on layer-specific feedback connectivity. To probe this question, we contrasted different patterns of feedback connectivity.

#### Constant feedback target.

First, we set up model variants augmented with a constant feedback target (final ReLU layer of convolution block 1) that received *short*-, *medium*-, or *long*-range feedback, for the short backbone and an additional *longer*-range feedback variant for the long backbone, as detailed in the Methods (see Experiment 2). The average difference in model performance between the first (t = 0) and the final (t = 15) for all feedback model variants was compared.

**Fig 2:**
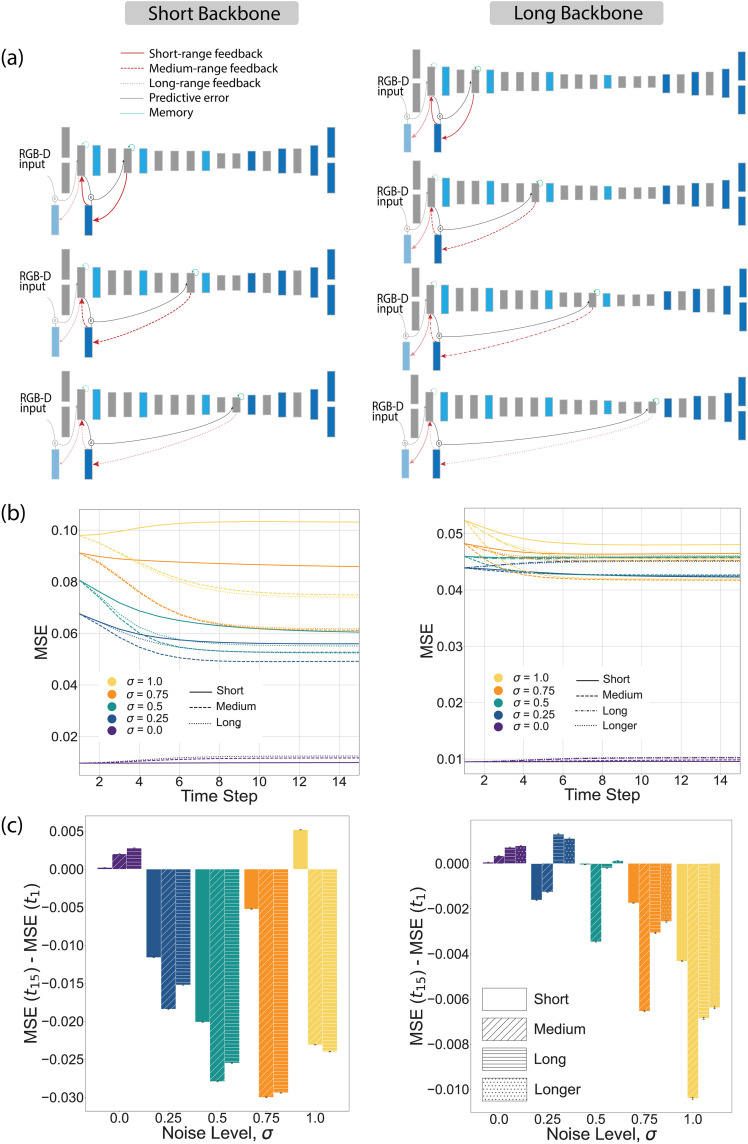
Layer-dependent effects of feedback with a uniform target. **(a)** Left panel: short feedforward model, augmented with *short*-range (first row; solid red arrow), *medium*-range (second row; dashed red arrow) and *long*-range (third row; dotted red arrow) feedback. Right panel: long feedforward model, augmented with *short*-range (first row; solid red arrow), *medium*-range (second row; dashed red arrow), *long*-range (third row; dash-dotted red arrow) and *longer*-range (fourth row; dotted red arrow) feedback. All effective feedback connections, pertaining to the second PCoder in each model variant, terminate at the same target layer: the final ReLU layer of convolution block 1. The first PCoder (translucent) does not represent a functional feedback connection. **(b)** Performance of feedback model variants across 15 predictive coding timesteps, on test dataset injected with varying levels of Gaussian noise (standard deviation, σ={0, 0.25, 0.5, 0.75, 1.0}). Left panel: short feedforward backbone. Right panel: long feedforward backbone. **(c)** The average difference in test performance of the feedback model variants, between the initial (t = 1) and final (t = 15) timestep, for each noise level. Black bars denote standard errors of mean. Left panel: short feedforward backbone. Right panel: long feedforward backbone.

For the short backbone, *medium*-range feedback confers the largest advantage on model performance on average under Gaussian noise (σ={0.25, 0.5, 0.75}), as seen in Fig 2b and 2c. A repeated measures ANOVA after Greenhouse-Geisser correction yielded a significant main effect of noise-dependent benefit of feedback on model performance (see Table 1) reflecting that performance declined with increasing noise, as expected. More importantly, there was a significant main effect of feedback distance (see Table 2). Post hoc comparisons using Tukey’s HSD test revealed significant differences in model performance between all pairs of feedback types with medium-range feedback being the most effective on average (see Table 3). Additionally, there was a significant interaction between feedback distance and noise level (see Table 4), indicating that most effective feedback distance depended on the noise-dependent benefit (*medium*-range feedback for intermediate levels of noise, *short*-range for zero noise, *long*-range for high levels of noise).

**Table 1:** Within Subjects Effects - Noise-dependent benefit. df: degrees of freedom for the effect (Residual gives the denominator df); F: F statistic; p: p-value; η²: partial eta squared (effect size).

Experiment			df	F	p	η^2^
**2**	Short backbone	Noise-dependent benefit	1.68	109649	<.001	0.454
		Residual	15772.36			
	Long backbone	Noise-dependent benefit	1.64	43410	<.001	0.426
		Residual	15398.52			
**3**	Short backbone	Noise-dependent benefit	1.56			
		Residual	14671.10	59300	<.001	0.652
	Long backbone	Noise-dependent benefit	2.71	24519	<.001	0.376
		Residual	25388.71			
**4**	Long backbone	Noise-dependent benefit	2.13	48395	<.001	0.520
		Residual	19937.27			

**Table 2:** Within Subjects Effects - Feedback distance (abstraction level). df: degrees of freedom for the effect (Residual gives the denominator df); F: F statistic; p: p-value; η²: partial eta squared (effect size).

Experiment			df	F	p	η^2^
**2**	Short backbone	Feedback distance	1.18	87764	<.001	0.194
		Residual	11072.25			
	Long backbone	Feedback distance	1.64	10542	<.001	0.080
		Residual	15429.00			
**3**	Short backbone	Feedback distance	1.10	5365	<.001	0.010
		Residual	10297.82			
	Long backbone	Feedback distance	2.61	2908	<.001	0.004
		Residual	24479.40			
**4**	Long backbone	Feedback abstraction level	1.35	13523	<.001	0.039
		Residual	12629.91			

**Table 3:** Post Hoc Comparisons - Feedback distance (abstraction level). Mean Difference (second level - first level), SE (standard error), df (degrees of freedom), t (t-statistic), and p_Tukey (Tukey-adjusted p-value).

Experiment		Comparison						
		Feedback distance (Exp 2–3); Feedback source abstraction (Exp 4)	Feedback distance (Exp 2–3); Feedback source abstraction (Exp 4)	Mean Difference	SE	df	t	p_tukey_
**2**	Short backbone	1	2	0.01316	4.05e-5	9381	324.5	<.001
			3	0.01195	4.21e-5	9381	284.1	<.001
		2	3	-0.00121	1.43e-5	9381	-85.0	<.001
	Long backbone	1	2	0.00274	1.97e-5	9381	138.88	<.001
			3	8.92e-5	2.56e-5	9381	3.48	0.003
			4	-1.42e − 4	2.42e-5	9381	-5.87	<.001
		2	3	-0.00265	1.57e-5	9381	-168.80	<.001
			4	-0.00288	1.56e-5	9381	-185.02	<.001
		3	4	-2.31e − 4	7.16e-6	9381	-32.33	<.001
**3**	Short backbone	1	2	9.75e-4	1.77e-5	9381	55.3	<.001
			3	-0.00200	2.55e-5	9381	-78.4	<.001
		2	3	-0.00297	4.01e-5	9381	-74.1	<.001
	Long backbone	1	2	2.80e-4	1.29e-5	9381	21.81	<.001
			3	2.91e-4	1.38e-5	9381	21.03	<.001
			4	-7.83e − 4	1.62e-5	9381	-48.41	<.001
		2	3	1.06e-5	1.06e-5	9381	1.01	0.745
			4	-0.00106	1.32e-5	9381	-80.30	<.001
		3	4	-0.00107	1.21e-5	9381	-88.67	<.001
**4**	Long backbone	1	2	-0.00157	1.54e-5	9381	-101.8	<.001
			3	-0.00223	1.68e-5	9381	-132.5	<.001
		2	3	-6.59e − 4	7.83e-6	9381	-84.2	<.001

**Table 4:** Within Subjects Effects - Feedback distance (abstraction level) x Noise-dependent benefit. df: degrees of freedom for the effect (Residual gives the denominator df); F: F statistic; p: p-value; η²: partial eta squared (effect size).

Experiment			df	F	p	η^2^
**2**	Short backbone	Feedback distance x Noise-dependent benefit	2.79	60426	<.001	0.176
		Residual	26216.36			
	Long backbone	Feedback distance x Noise-dependent benefit	4.94	5272	<.001	0.057
		Residual	46387.80			
**3**	Short backbone	Feedback distance x Noise-dependent benefit	1.94	13862	<.001	0.030
		Residual	18161.08			
	Long backbone	Feedback distance x Noise-dependent benefit	7.35	1884	<.001	0.009
		Residual	68946.23			
**4**	Long backbone	Feedback abstraction level x Noise-dependent benefit	5.61	1290	<.001	0.008
		Residual	52603.38			

Similarly, in the case of the long backbone, *medium*-range feedback showed on average the greatest robustness to noise (σ={0.5, 0.75, 1.0}), as shown in Fig 2b and 2c. A Greenhouse-Geisser corrected repeated measures ANOVA showed significant main effects of noise level (see Table 1), and feedback distance (see Table 2), where Tukey’s HSD test confirmed that *medium*-range feedback was most effective by establishing significant differences between various pairs of feedback types (see Table 3). Once again, the interaction between feedback distance and noise-dependent feedback benefit was also significant (see Table 4), indicating that *medium*-range feedback was most effective for intermediate and high levels of noise whereas *short*-range feedback was more effective for zero and low levels of noise.

#### Constant feedback source.

**Fig 3:**
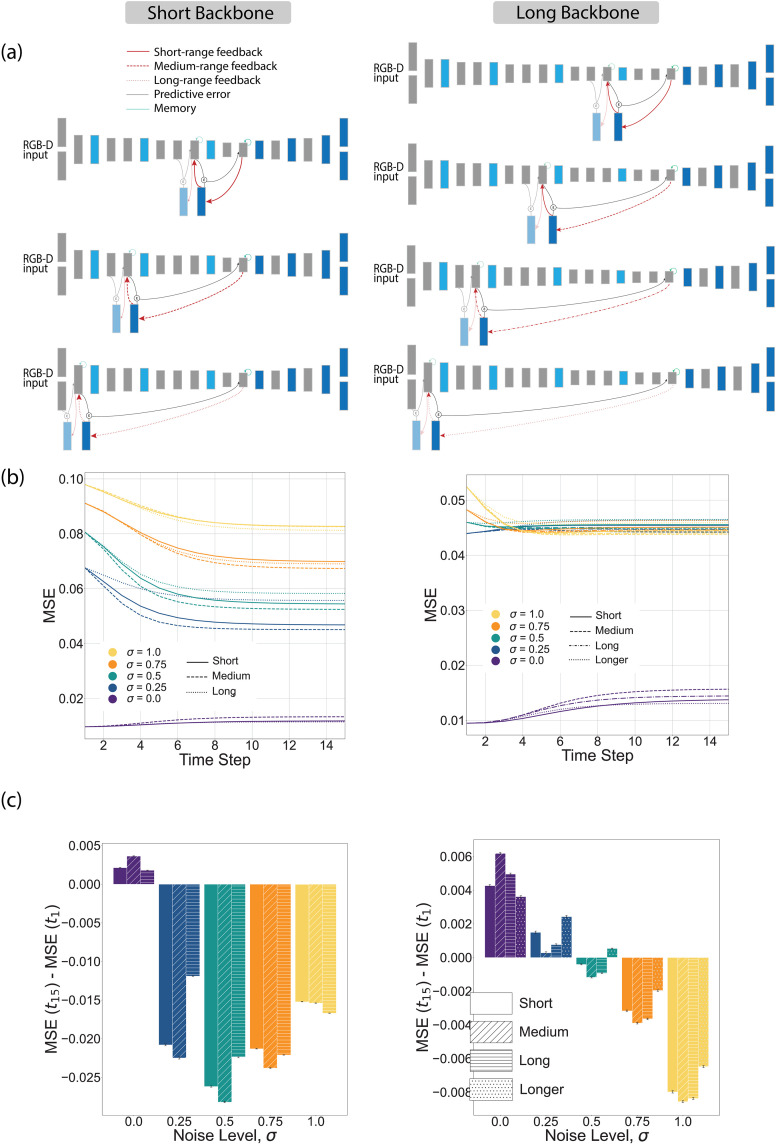
Layer-dependent effects of feedback with a uniform source. **(a)** Left panel: short feedforward model, augmented with *short*-range (first row; solid red arrow), *medium*-range (second row; dashed red arrow) and *long*-range (third row; dotted red arrow) feedback. Right panel: long feedforward model, augmented with *short*-range (first row; solid red arrow), *medium*-range (second row; dashed red arrow), *long*-range (third row; dash-dotted red arrow) and *longer*-range (fourth row; dotted red arrow) feedback. All effective feedback connections, pertaining to the second PCoder in each model variant, originate at the same source layer; short feedforward backbone: final ReLU layer of convolution block 4; long feedforward backbone: final ReLU layer of convolution block 5. The first PCoder (translucent) does not represent a functional feedback connection. **(b)** Performance of feedback model variants across 15 predictive coding timesteps, on test dataset injected with varying levels of Gaussian noise (standard deviation, σ={0, 0.25, 0.5, 0.75, 1.0}). Left panel: short feedforward backbone. Right panel: long feedforward backbone. **(c)** The average difference in test performance of the feedback model variants, between the initial (t = 1) and final (t = 15) timestep, for each noise level. Black bars denote standard errors of mean. Left panel: short feedforward backbone. Right panel: long feedforward backbone.

Secondly, we compared model performance under adversarial noise, when the feedback connections originated from the same source layer across all model variants (for details of the model architecture, see Experiment 3 in Methods). Model variants were evaluated after timestep t=15. Once again for the short backbone we found *medium*-range feedback was most ameliorative, when presented with stimuli injected especially with intermediate levels of Gaussian noise (σ={0.25, 0.5, 0.75}), as shown in Fig 3b and 3c. A repeated measures ANOVA with Greenhouse-Geisser correction demonstrated a significant main effect of noise-dependent benefit of feedback (see Table 1). More importantly, we observed a significant main effect of feedback distance on model performance (see Table 2), with *medium*-range feedback yielding the greatest benefit on average. In detail, Tukey’s HSD test revealed significant differences between various pairs of feedback types (see Table 3). Finally, there was a significant interaction between feedback distance and noise-dependent benefit (see Table 4), indicating that *medium*-range feedback was optimal for intermediate levels of noise whereas *long*-range feedback was more ameliorative for high and zero levels of noise*.*

Similar effects of feedback connectivity on model performance were observed in the case of the long backbone, Fig 3b and 3c. The network augmented with *medium*-range feedback was most resilient to all levels of additive noise tested, except for zero noise (σ={0.25, 0.5, 0.75, 1.0}). A repeated measures ANOVA with Greenhouse-Geisser correction showed significant main effects of noise-dependent feedback benefit (see Table 1), and feedback distance (see Table 2). Post hoc comparisons using Tukey’s HSD test revealed significant differences between the various pairs of feedback types (see Table 3). These results are consistent with *medium*-range feedback being most beneficial across noise levels on average. Furthermore, a significant interaction between feedback distance and noise-dependent benefit of feedback (see Table 4) reflected that *medium*-range feedback was optimal for most noise levels except zero noise.

### Proximal neural feedback loops improve robustness to noise

**Fig 4:**
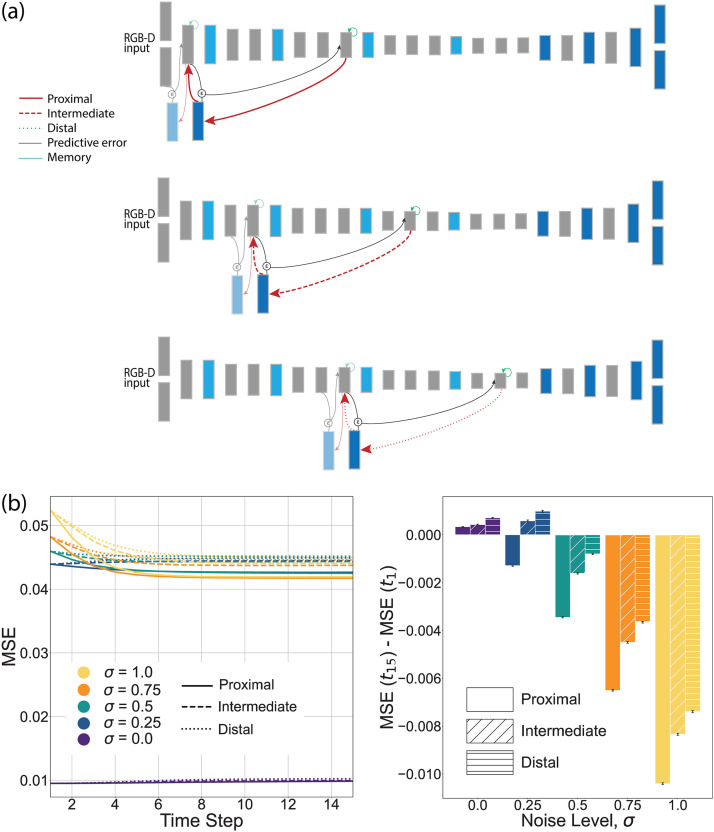
Effect of feedback source abstraction level. **(a)** Long feedforward model, augmented with *medium*-range feedback connections (pertaining to the second PCoder in each model variant), originating at layers *proximal* (first row; solid red arrow), *intermediate* (second row; dashed red arrow), or *distal* (third row; dotted red arrow) to the input layer. The first PCoder (translucent) does not represent a functional feedback connection. **(b)** Left panel: performance of feedback model variants across 15 predictive coding timesteps, on test dataset injected with varying levels of Gaussian noise (standard deviation, σ={0, 0.25, 0.5, 0.75, 1.0}). Right panel: the average difference in test performance of the feedback model variants, between the initial (t = 1) and final (t = 15) timestep, for each noise level. Black bars denote standard errors of mean.

Having established the precedence of *medium*-range neural feedback loops in two distinct neural network architectures, we investigated if the performance-enhancing effects of such feedback were sensitive to the level of abstraction of the feedback source, i.e., feedback originating from early vs. late areas in the dorsal visual stream. Using the long backbone architecture, we set up three different model variants, augmented with *medium*-range feedback connections, originating at *proximal*, *intermediate* or *distal* layers relative to the input (see Experiment 4 in Methods), which were then presented with input images corrupted with Gaussian noise. Feedback most *proximal* to the input layer, had the greatest performance-enhancing effect, as seen in Fig 4b. A repeated measures ANOVA with Greenhouse-Geisser correction examined the effects of feedback source abstraction level, as well as noise level, on the change in performance of the model between timestep t=0 and timestep t=15. The analysis identified significant main effects of noise-dependent benefit of feedback (see Table 1), as well as feedback abstraction level (Table 2). The latter effect reflected proximal feedback to be optimal as shown with post hoc comparisons using Tukey’s HSD test indicating significant differences between various pairs of feedback abstraction levels (see Table 3). Moreover, the interaction between abstraction and noise level was significant (see Table 4) due to the influence of abstraction level being less prominent for zero noise than for other noise levels.

### Evaluation under a robotic grasp-detection metric

**Fig 5:**
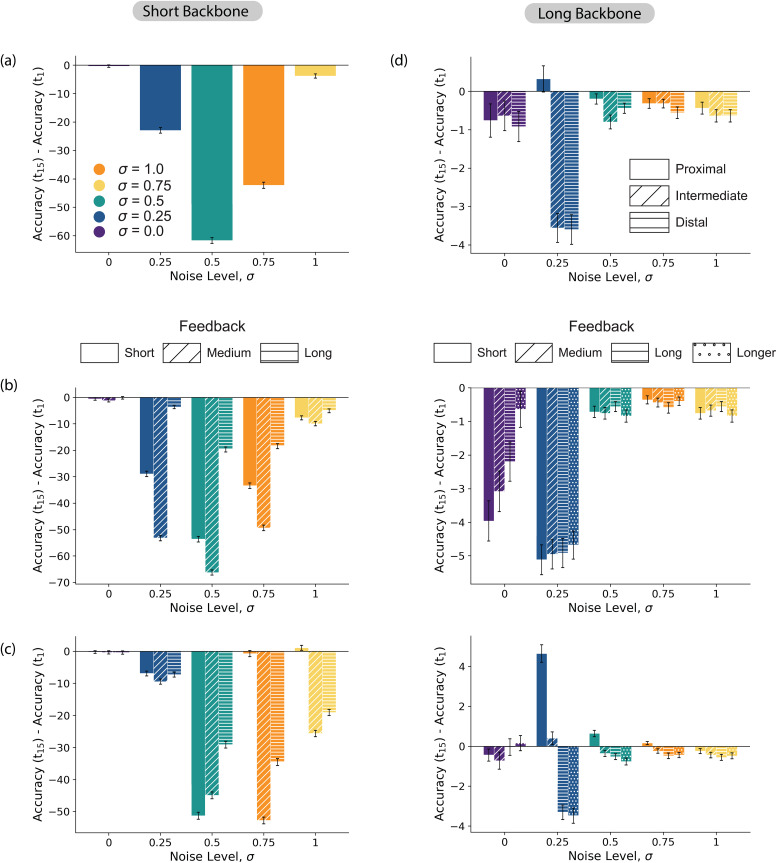
Evaluation under a robotic grasp detection metric. The average difference in IoU-based accuracy of feedback model variants, between the initial (t = 1) and final (t = 15) predictive coding timestep, evaluated on the test dataset injected with varying levels of additive Gaussian noise (standard deviation, σ={0, 0.25, 0.5, 0.75, 1.0}). **(a)** Experiment 1. Short baseline augmented with symmetric feedback. **(b)** Left panel: Experiment 2. Short baseline augmented with *short*-, *medium*- and *long*-range asymmetric feedback, terminating at a uniform feedback target. Right panel: Long-baseline augmented with *short*-, *medium*-, *long*- and *longer*-range asymmetric feedback, terminating at a uniform feedback target. **(c)** Left panel: Experiment 3. Short baseline augmented with *short*-, *medium*- and *long*-range asymmetric feedback, originating at a uniform feedback source. Right panel: Long-baseline augmented with *short*-, *medium*-, *long*- and *longer*-range asymmetric feedback, originating at a uniform feedback source. **(d)** Experiment 4. Long-baseline augmented with *medium*-range feedback, originating at a *proximal*, *intermediate* or *distal* source. Black bars denote standard errors of mean.

To complement our representation-level analyses, we additionally evaluated all model variants using the rectangle-based intersection-over-union (IoU) metric conventionally used for benchmarking robotic grasp detection [30,31], as adopted in the Jacquard dataset [29]. Across all experiments (feedback path length and feedback source abstraction), IoU-based accuracy did not show a clear monotonic trend with either feedback distance or source abstraction level, in contrast to the MSE-based results reported above. We even find evidence that feedback impacted this robotic grasping measure, which is contrary to the MSE-based results reported above. The IoU results across feedback variants and noise levels are summarized in Fig 5. We attribute this divergence to a difference in what the two metrics measure. MSE on the dense parametric map captures graded changes in the fidelity of the model’s distributed grasp representation. This is what we reason comes closest to mechanisms in the primate cortex given neurophysiological data [32]. By contrast, the IoU metric was designed to evaluate whether a single committed gripper pose, extracted by a discrete decoding step, sufficiently matches an annotated rectangle for downstream robotic execution. The latter collapses graded representational changes into a binary success/failure for one selected pose, at a granularity that is mismatched to the visual-representational stage our model is intended to capture (see Discussion).

## Discussion

In the present study, we investigated the contribution of neural feedback to visual processing for computing grasp parameters by utilizing convolutional neural network models that had been augmented with predictive feedback and that were trained to compute grasp positions for real-world objects. After establishing an ameliorative effect of symmetric feedback on grasp detection from noisy images, we characterized the performance effects of asymmetric feedback, similar to that observed in the cortex. We found that the performance-enhancing effect of predictive coding under adverse conditions was optimal, across a range of noise levels, for *medium*-range asymmetric feedback. Moreover, this effect was most prominent when *medium*-range feedback originated at a level of representational abstraction that was proximal to the input layer, in contrast to more distal layers.

Prior computational work has demonstrated that symmetric feedback, originating from the immediate next higher area in the cortical hierarchy, helps suppress irrelevant noise to enhance performance in an object classification task [17,18]. Importantly, our work extends the role of predictive feedback in noise-cleanup to the more general function of visual processing for grasp prediction. This involves progressively denoising the feature representations during the test phase, essentially by projecting the representations onto the learned manifolds in the representational space of the trained model. Consistent with this idea, iterative predictive coding dynamics worsen the model’s performance when presented with clean images. Even if the input image is clean, the decoder is not a perfect inverse of the forward path, so $d_{n}(t)$ representation will not exactly match $e_{n-\text{1}}(t)$. The predictive coding update therefore still sees a non-zero error map and will apply corrections at each time step. The corrections minimize a reconstruction loss (the difference between $d_{n}$ and $e_{n-\text{1}}$), which can iteratively bias a solution toward an average prototype representation (or manifold), nudging it away from the task-optimal representation found during the first feedforward pass.

However, accumulating neurophysiological evidence has suggested that cortical feedback pathways are not strictly reciprocal [20] and may traverse multiple areas at different latencies [33,19]. By systematically comparing different feedback path lengths and levels of representational abstraction, we illustrated that too short a feedback loop may lack the broader contextual information needed to mitigate noise effectively, while longer loops may carry overly abstract predictions. We propose that *medium*-range feedback marks a unique sweet spot, putatively incorporating an optimal neural transmission delay and an informative level of abstraction of the feedback source. Moreover, our results suggest that *medium*-range feedback benefits noise clean-up the most when it originates from layers that are proximal to the input, suggesting that relatively early representations are most effective in removing noise. Future work will also investigate the generalizability of the benefits of *medium*-range feedback to different task objectives, such as object-recognition. We expect certain types of asymmetric feedback to be more beneficial than others when viewing noisy visual stimuli, reflecting either alignment or dissimilitude between the dorsal visual (action-guided vision) and ventral visual (object recognition) streams in the brain.

Of note is the observed U-shaped effect of noise-dependent performance benefit of predictive feedback for the short backbone experiments (Figs 2C, left, and 3C, left) vs. a linear decrease observed for the long backbone models (Figs 2C, right; 3C, right; and 4B, right), across the five discrete levels of additive Gaussian noise σ={0, 0.25, 0.5, 0.75, 1.0} that were tested. We speculate that the linear trend seen for the long backbone is the arm of a shifted, wider U-shape reflecting robustness to noise perturbation and recovery for the higher-dimensional (in terms of parametric space) long backbone models. The hypothesized U-shaped may emerge if the long backbone model variants are evaluated on higher levels of additive Gaussian noise, e.g., σ={1.25, 1.5, 1.75, …}.

We only tested the ameliorative effect of PC on visual stimulus corruption in the form of Gaussian noise, which prior works have reported to be particularly amenable to this effect [34]. However, the Predify framework has been previously tested on 19 ImageNet-C corruptions [35] and observed benefits for 16 of them (in particular, Gaussian noise, shot noise, impulse noise and speckle noise), indicating robustness is not limited to a single type of noise [17]. While we did not explicitly test for more “naturalistic” noise (e.g., occlusion), we speculate that long- or longer-range feedback may be most advantageous for such structured disruptions that require integrating broader (global) context or inferring missing parts.

Together our findings suggest a previously uncharted role of intermediate cortical areas along the dorsal stream, e.g., roughly similar to area V6A in the primate brain, as a source of predictive feedback. V6A is primarily involved in encoding visual processing upstream of grasping behaviors [36]. It plays a key role in guiding prehension movements [36]. Importantly, V6A integrates both visual inputs and somatosensory feedback from the upper limbs [37]. Our results indicate that V6A might be at a strategically optimal position to exert dynamic top-down influence onto early visual areas during visuomotor processing, thus constituting an important hypothesis for future research.

A related point concerns how performance is measured in a model of this kind. The robotics literature has standardized the rectangle-based intersection-over-union (IoU) metric [30,31], adopted in the Jacquard benchmark [29], as the standard for evaluating robotic grasp-detection systems. This metric was designed to assess if a model’s output sufficiently matches any annotated grasp for successful execution by a parallel-jaw end effector. It is, by construction, a metric of downstream robotic success rather than a metric of the visual representation that precedes pose commitment. Our model, in contrast, is intended as a model of that earlier visual stage. Neurophysiological evidence indicates that intermediate- and late-stage areas of the dorsal grasping network encode object affordances and a distribution over potential grasps rather than a single committed rectangle: V6A neurons are modulated by object affordances during visual presentation, before grasp execution [32], and grip configurations can be decoded from V6A populations during the delay period preceding movement [38], consistent with the more general framework in which parietal cortex represents the space of available affordances and selection occurs downstream [39,40]. The dense parametric grasp map produced by our network is intended as the model analog of such a distributed representation, and our central scientific question concerns the *fidelity* of this representation under noise, not the success rate of any committed pose extracted from it. MSE on the dense map measures this representational fidelity directly, whereas IoU collapses graded representational changes into a binary success/failure for a single decoded pose, as an agreed upon benchmark in robotics. When we evaluated our models with the standard IoU metric (Fig 5), we observed no clear monotonic trend with feedback path length or source abstraction, as would be expected if a discrete decoding step is insensitive to the kind of distributional refinement predictive feedback induces. While IoU serves as a useful point of comparison within the grasp-detection literature, it does not capture all aspects of grasp performance. Future work could benefit from incorporating more ecologically valid evaluation criteria, such as grasp success (e.g., object retention) alongside task-dependent optimization considerations. For example, grasps near the center of mass may be optimal for transport, whereas grasps farther from it may be required for tool use. The effects of feedback on such criteria warrant further investigation in future studies.

As for studying the effects of feedback on representative models, it is intriguing to note that feedback arising from proximal layers of the network is more beneficial than feedback coming from distal layers. Interestingly, the opposite pattern of effectiveness was reported for simulated feedback emulating attentional effects in a VGG16 network [41]. Lindsay and Miller [41] found that attentional effects were most noticeable when injected into more distal layers. This difference in proximal vs. distal efficacy of feedback is not explained by structural differences in the feedforward networks given that the current study used the same long backbone employed previously [41]. The difference in efficacy could be due to task differences. However, Lindsay and Miller [41] observed the same distal benefits for a range of tasks. Instead, the proximal vs. distal differences might, and probably do, come from the disparity in feedback. Whereas in the present study we implemented predictive feedback signals, Lindsay and Miller [41] simulated attentional feedback according to the feature similarity gain model [42,43] where neuronal activity changes as a function of similarity of a neuron’s tuning to an attended feature. Whether this particular kind of attentional modulation or more generally the function of attention as a form of task-relevant resource allocation in contrast to task-independent predictive coding [44] can explain the differences between proximal vs. distal effectiveness should be explored in the future.

We also note that the current choice of a 2-step training regime, which optimizes the feedforward and feedback connections separately, may not be biologically realistic. However, this approach was selected to keep computation tractable. Joint optimization requires unrolling a deep backbone (e.g., VGG-like in our case) over many timesteps (we currently analyze up to t=15), which is memory-prohibitive on available hardware. While a more compact neural network could make joint training permissible, the reduced depth of the backbone may severely limit testing of varying feedback path lengths. Future efforts can leverage memory-saving strategies to directly compare separate versus joint training across feedback distances and tasks.

We deem important to highlight that the feedback models may not capture the full complexity of a biological system where multiple feedback connections may be operative concurrently. For interpretability, however, we evaluated models with a single functional feedback connection (Experiments 2–4), with systematically varying path lengths and source abstraction levels. This design choice isolates the causal effect of a given feedback loop without confounds from loop-loop interactions, producing a clear mapping between feedback distance and perceptual robustness. We view our single-loop findings as components of a cortical regime where multiple feedback pathways likely operate simultaneously across ranges. Extending this framework to multi-loop models, potentially with learned gating or joint optimization where computationally feasible, will be an important future direction.

In conclusion, our simulations show that introducing biologically realistic asymmetric predictive feedback improves model robustness to noisy visual stimuli in a neural network model optimized for grasp detection. Our findings highlight the possible functional significance of mid-tier dorsal stream areas (e.g., V6A) that lie between low-level (V1) and high-level (parietal) representations, suggesting these areas might serve as ideal “predictors” for early visuomotor processing.

## Methods

### Neural network architecture

We first trained two versions of a feedforward model, a short and a long backbone, on the task of grasp point generation. As a second step we then augmented the networks with feedback connections (feedback model) that conveyed predictive signals.

#### Short feedforward model.

In detail, for step 1 we obtained the architecture of the short backbone model by modifying a custom convolutional neural network (CNN) architecture (see Fig 1a) into which we inputted photographic images with 4 channels (aside from red, green, and blue there was a depth channel, RGB-D, as an approximation of stereovision, that is known to be relevant for grasp performance [45]). The RGB-D images were passed into an input layer with two arms, specialized for receiving RGB and depth channels, respectively. The backbone contained a ‘feature compression’ module consisting of 4 convolution blocks that were connected through maximum pooling (MaxPool) layers to attain dimensionality reduction and invariant feature extraction [46]. That is, each convolution block consisted of two or three convolution (Conv) layers, each of which was sandwiched together with a batch normalization (BN) and a rectified linear unit (ReLU) activation layer. Next, we added a ‘feature expansion’ module, composed of Conv and ConvTranspose2d layers [47] that upscaled the feature maps back to the same spatial dimensions as the input (i.e., the entire network had an autoencoder style architecture). The output layer was a regression head that generated a 6-channel array of pixel-wise maps, each corresponding to one of five grasp parameters required for grasping (grasp centre coordinates (x, y); orientation; grasp opening; gripper size) and a grasp quality score map, similar to [48]. The Jacquard dataset [29] provides annotations as a list of discrete grasp-candidate rectangles per object, which we transformed into this dense pixel-wise map representation for training (see Methods for details of the transformation). The sole purpose of the feature expansion module together with its map-style output was not to attain biological realism but to facilitate interpolation of the infinite number of correct grasp solutions from the finite set of ground truth labels (e.g., [48]). This format of the model output not only captures the complete solution space of mutually exclusive grasps, instead of limited pointillistic solutions, but also facilitates training of the feedforward network by eliminating the need for computationally expensive postprocessing of the model output that is needed to achieve spatial correspondence between discrete predicted and ground-truth grasps. The network architecture of the short backbone is detailed in Table 5. Beyond these practical considerations, the dense map representation is also conceptually aligned with the model’s intended role as an analog of cortical grasp-relevant visual representations: neurophysiological evidence indicates that intermediate dorsal-stream areas such as V6A encode a distribution over potential grasps rather than a single committed rectangle [32,38], making a dense parametric map a more appropriate output format than a discrete rectangle list for a model of this visual stage.

**Table 5:** Neural network architecture of the short and long feedforward models. Layer-wise architecture of the feedforward models is described. All layers were implemented in PyTorch. The Conv2d (output channels, kernel size, stride) and ConvTranspose2d (output channels, kernel size, stride) refer to convolutional and transpose convolutional layers, respectively. The MaxPool (kernel size, stride) layer implements feature pooling. BatchNorm2d: batch normalization; ReLU: rectifying linear unit; Tanh: hyperbolic tangent; Sigmoid: sigmoid function.

Module		Layers	Short	Long
Input	RGB	Conv2d (64, 3, 1)	•	•
	Depth	Conv2d (64, 3, 1)	•	•
Feature compression		BatchNorm2d | ReLU	•	•
	Conv block 1	Conv2d (64, 3, 1) | BatchNorm2d | ReLU	•	•
		MaxPool (2, 2)	•	•
	Conv block 2	Conv2d (128, 3, 1) | BatchNorm2d | ReLU	•	•
		Conv2d (128, 3, 1) | BatchNorm2d | ReLU	•	•
		MaxPool (2, 2)	•	•
	Conv block 3	Conv2d (256, 3, 1) | BatchNorm2d | ReLU	•	•
		Conv2d (256, 3, 1) | BatchNorm2d | ReLU	•	•
		Conv2d (256, 3, 1) | BatchNorm2d | ReLU	•	•
		MaxPool (2, 2)	•	•
	Conv block 4	Conv2d (256, 3, 1) | BatchNorm2d | ReLU	•	
		Conv2d (256, 3, 1) | BatchNorm2d | ReLU	•	
		Conv2d (512, 3, 1) | BatchNorm2d | ReLU		•
		Conv2d (512, 3, 1) | BatchNorm2d | ReLU		•
		Conv2d (512, 3, 1) | BatchNorm2d | ReLU		•
		MaxPool (2, 2)		•
	Conv block 5	Conv2d (512, 3, 1) | BatchNorm2d | ReLU		•
		Conv2d (512, 3, 1) | BatchNorm2d | ReLU		•
		Conv2d (512, 3, 1) | BatchNorm2d | ReLU		•
Feature expansion		ConvTranspose2d (256, 3, 2) | ReLU		•
		Conv2d (256, 5, 1) | ReLU		•
		ConvTranspose2d (128, 3, 2) | ReLU	•	•
		Conv2d (128, 5, 1) | ReLU	•	•
		ConvTranspose2d (64, 3, 2) | ReLU	•	•
		Conv2d (128, 5, 1) | ReLU	•	•
		ConvTranspose2d (64, 3, 2) | ReLU	•	•
Output	Grasp	ConvTranspose2d (5, 3, 1) | Tanh	•	•
	Confidence	ConvTranspose2d (1, 3, 1) | Sigmoid	•	•

#### Long feedforward model.

The long backbone once again had a compression module followed by an expansion module similar to the short backbone, except, here the compression module comprised of a VGG16 model [46] with 5 convolution blocks where each convolution block contained two or three Conv layers with ReLU activation layers, followed by a MaxPool layer, and BN layers interspersed between each Conv and ReLU layer. Just like before the model architecture was modified with a two-arm input layer to receive 4-channel RGB-D input images. Also, the classification head of the canonical VGG16 architecture was replaced with the same regression head as the short backbone model to return the required parametric map output (see Table 5 for details).

### Predictive coding dynamics for feedback models

After training the feedforward backbone models, we augmented them with generative feedback connections, adapting the PyTorch *Predify* library [17] with some modifications that we made to the original code to introduce custom feedback connectivity. The library is designed to add predictive coding dynamics to existing deep neural networks. The predictive coding framework posits that the brain maintains an internal model of the world to actively predict sensory inputs [49]. In this hierarchical model, higher areas generate predictions for lower areas, and discrepancies between predicted and actual inputs (prediction errors) are used to update and refine the higher-layer representations. This iterative process allows the network to minimize prediction errors and enhance perception of sensory information. For example, CNNs have been recently combined with feedback mechanisms for robust object perception [17,18].

In the present study, we selected N encoding modules, $e_{n}$ (n∈{1,2,…,N} with $N{\in Z}^{+})$ from the network backbone of the feedforward model and added N corresponding decoding modules, $d_{n}$. An encoding module, $e_{n}$, and the corresponding decoding module, $d_{n}$, collectively constituted a *PCoder*. Each $e_{n}$ (except for e_*1*_) consisted of the MaxPool layer of a given convolution block and the Conv/BN/ReLU layers of the subsequent convolution block. The output of $e_{n}$ was then passed through a feedback layer (transpose convolution layer) $d_{n}$ that predicted the output of the last Conv/BN/ReLU layer of the previous convolution block (target). To this end, each $d_{n}$ consisted of a ConvTranspose2d layer that upscaled the input feature map to match the spatial dimensions of the target with feedback weights connecting module n+1 to module n being denoted by $\overset{b}{\underset{n+\text{1},n}{W}}$. This way, when an input image initially activated all encoding modules through a feedforward pass, over subsequent successive recurrent iterations (timesteps t), both the decoding and encoding module representations were updated using the following equations:


$$\begin{array}{c} \;\overset{t}{\underset{n}{d}}=\;\overset{b}{\underset{n+\text{1},n}{W}}\overset{t}{\underset{n}{e}}\; \end{array}$$ (1)

$$\begin{array}{c} \;\overset{t+\text{1}}{\underset{n}{e}}=\;{e_{n}(\beta }_{n}\overset{t+\text{1}}{\underset{n-\text{1}}{e}})+\;\lambda_{n}\overset{t}{\underset{n+\text{1}}{d}}+\gamma_{n}\overset{t}{\underset{n}{e}}-\;\alpha_{n}\nabla \overset{t}{\underset{n}{\epsilon }} \end{array}$$ (2)

Here, $\beta_{n}$, $\lambda_{n}$, $\gamma_{n}$ (with $\beta_{n}+\lambda_{n}+\gamma_{n}=$ 1) and $\alpha_{n}$ served as balancing coefficients for the feedforward, feedback, recurrence and error-correction terms, respectively. The recurrence term, $\gamma_{n}$, functioned as a memory buffer for retaining the encoding representation at the current timestep. The reconstruction error at module n−1, denoted $\epsilon_{n-\text{1}}(t)$, was defined as the mean squared error (MSE) between the feedforward representation $e_{n-\text{1}}(t)$ and the predicted reconstruction $d_{n-\text{1}}(t)$ at that timestep. The MSE is calculated as a mean of the elementwise differences between $e_{n-\text{1}}(t)$ and $d_{n-\text{1}}(t)$, i.e., a scalar value corresponding to each timestep. A gradient of the MSE with respect to $e_{n-\text{1}}(t)$, $\nabla \overset{t}{\underset{n}{\epsilon }}$, is backpropagated to $e_{n-\text{1}}(t)$ as an error signal. The feedforward and feedback weights remained frozen across iterations.

#### Feedback models.

Modifications were made to the original code from [17] to introduce custom feedback connectivity to the forward models. For the purposes of this study, each MaxPool layer and the Conv/BN/ReLU layers of the subsequent convolution block are treated as the model equivalent of *a single brain area*. *Short*-, *medium*-, *long*- and *longer*-range feedback projections skip over one, two, three and four virtual brain areas, respectively.

The effects of feedback path length and representational abstraction of the feedback source on noise-cleanup were tested through a nested experimental design. Experiments 2–3 explored the effects of feedback path length (*short*-, *medium*-, and *long*-range feedback connections added to a short backbone model, and *short*-, *medium*-, *long*- and *longer*-range feedback added to a long backbone model) on model performance when presented with noisy visual stimuli. Experiment 4 further investigated the denoising effect of *medium*-range feedback, originating at varying levels of representational abstraction (feedback source is *proximal*, *intermediate* or *distal* to the input layer) in a long backbone model.

***Experiment 1*:** To test whether predictive coding aids not only object classification [17,18] but also visual processing for computing grasp parameters, the network backbone of the short backbone model was augmented with 4 consecutive PCoders, similar to the feedback connectivity pattern attempted in [17]. The feedback connectivity for Experiment 1 is shown in Fig 1a.

***Experiment 2*:** In order to identify the most effective source of feedback, feedback connections were implemented with a consistent feedback target layer and varying feedback source layers across model variants. Specifically, for each feedback type, 2 PCoders were designed. For Experiment 2, $e_{\text{1}}\;$was kept fixed as the first convolution block, across all feedback model variants. Notably, the feedback target layer for $e_{\text{1}}$ was the input layer of the network; the input representations were configured to be static and were not updated by iterative recurrent mechanisms. The associated feedback loop was merely an appendage, which had no functional effect but was required in the model architecture for technical reasons. Additionally, the feedback source layer of $e_{\text{2}}$ did not receive any recurrent feedback. Therefore, all model variants of feedback connectivity effectively contained a single functional loop of feedback, shown as red lines in Fig 2, for controlled comparisons between different feedback types.

*Short backbone.* Three variants of feedback connectivity were compared: *short-*range, *medium-*range and *long-*range feedback, as shown in Fig 2a. $e_{\text{1}}$ comprised of the Conv/BN/ReLU layers of convolution block 1 for all model variants. $e_{\text{2}}$ comprised of the following: the MaxPool layer of block 1 and the Conv/BN/ReLU layers of block 2 (*short-*range); the MaxPool layers of blocks 1 and 2, and the Conv/BN/ReLU layers of blocks 2 and 3 (*medium-*range); the MaxPool layers of blocks 1, 2 and 3, and the Conv/BN/ReLU layers of blocks 2,3 and 4 (*long-*range). The *short*-, *medium*- and *long*-range feedback connections are analogous to cortical feedback loops cascading over one, two and three brain areas, respectively.

*Long backbone.* Four variants of feedback connectivity were compared: *short-*range, *medium-*range, *long-*range and *longer-*range feedback, illustrated in Fig 2a. Similar to the short backbone, $e_{\text{1}}$ comprised of the Conv/BN/ReLU layers of convolution block 1 for all model variants. $e_{\text{2}}$ comprised of the following: the MaxPool layer of block 1 and the Conv/BN/ReLU layers of block 2 (*short-*range); the MaxPool layers of blocks 1 and 2, and the Conv/BN/ReLU layers of block 2 and 3 (*medium-*range); the MaxPool layers of blocks 1,2 and 3, and the Conv/BN/ReLU layers of block 2, 3 and 4 (*long-*range); the MaxPool layers of blocks 1, 2, 3 and 4, and the Conv/BN/ReLU layers of block 2, 3, 4 and 5 (*longer-*range). The *short*-, *medium*-, *long*- and *longer*-range feedback connections are analogous to cortical feedback pathways skipping over one, two, three or four brain areas, respectively.

***Experiment 3*:** To further isolate any effects of the feedback target layer, the feedback source layer was kept constant, while varying feedback target layers across model variants. Again, 2 PCoders were introduced into the feedforward network. Crucially, the feedback connection emanating from $e_{\text{2}}$ was the only functional feedback loop. For all model variants, $e_{\text{1}}$ spanned a single Conv layer that immediately precedes $e_{\text{2}}$.

*Short backbone.* Three variants of feedback connectivity were compared: *short-*range, *medium-*range and *long-*range feedback. The feedback source layer of $e_{\text{2}}$ was kept fixed as the final ReLU layer of convolution block 4 for all feedback variants. $e_{\text{2}}$ comprised of the following: the MaxPool layer of block 3 and the Conv/BN/ReLU layers of block 4 (*short-*range); the MaxPool layers of blocks 2 and 3, and the Conv/BN/ReLU layers of blocks 3 and 4 (*medium-*range); the MaxPool layers of blocks 1,2 and 3, and the Conv/BN/ReLU layers of blocks 2, 3 and 4 (*long-*range). These model variants of feedback connectivity are shown in Fig 3a.

*Long backbone.* Four variants of feedback connectivity were compared: *short-*range, *medium-*range, *long-*range and *longer-*range feedback. The feedback source layer of $e_{\text{2}}$ was maintained as the final ReLU layer of convolution block 5 for all feedback variants. $e_{\text{2}}$ comprised of the following: the MaxPool layer of block 4 and the Conv/BN/ReLU layers of block 5 (*short-*range); the MaxPool layers of blocks 3 and 4, and the Conv/BN/ReLU layers of blocks 4 and 5 (*medium-*range); the MaxPool layers of blocks 2, 3 and 4, and the Conv/BN/ReLU layers of blocks 3, 4 and 5 (*long-*range); the MaxPool layers of blocks 1, 2, 3 and 4, and the Conv/BN/ReLU layers of blocks 2, 3, 4 and 5 (*longer-*range). Model variants of feedback connectivity are shown in Fig 3a.

***Experiment 4*:** The effect of the abstraction level of the feedback source on model performance was also investigated. *Medium-*range feedback (i.e., the most effective feedback distance in Experiments 2 and 3) was implemented at three different levels of abstraction along the long backbone: *proximal*, *intermediate* and *distal* feedback loops. The naming convention is in relation to the distance from the input layer of the network. For each level of source abstraction, 2 PCoders were integrated into the network backbone. Like Experiments 2 and 3, feedback emerging from $e_{\text{2}}$ was the only operant feedback connection, whereas the feedback loop across $e_{\text{1}}$ traversed a single layer and had no predictive function.

For the three model variants, $e_{\text{2}}$ comprised of the MaxPool layers of blocks 1 and 2, and the Conv/BN/ReLU layers of blocks 2 and 3 (*proximal*); the MaxPool layers of blocks 2 and 3, and the Conv/BN/ReLU layers of blocks 3 and 4 (only the first two Conv/BN/ReLU layers of block 4 were included in order to implement *medium-*range feedback distance) (*intermediate*); the MaxPool layers of blocks 3 and 4, and the Conv/BN/ReLU layers of blocks 4 and 5 (only the first two Conv/BN/ReLU layers of block 5 were included in order to maintain *medium-*range feedback distance) (*distal*). The model variants of *medium*-range feedback that were implemented are detailed in Fig 4a.

### Model training and hyperparameters

Both, short and long backbone models were initialized with random weights (Xavier initialization) and trained on the task of grasp parameter estimation. Training involved error back-propagation and gradient descent to minimize the mean squared error (MSE) loss for the pixelwise regression output. Specifically, the network outputs a six-channel (center pixel coordinates (x, y), orientation, dimensions (pixel width, pixel height) of the grasp and grasp-quality score) pixel-wise map ${\hat{Y}}_{t}\in R^{\text{6}\times H\times W}$at timestep t. Performance is the mean-squared error (MSE) between ${\hat{Y}}_{t}$ and the ground-truth map Y:


$$\begin{array}{c} {\text{MSE}}_{t}=\frac{\text{1}}{\text{6}HW}\sum_{c=\text{1}}^{\text{6}}\;\sum_{i=\text{1}}^{H}\;\sum_{j=\text{1}}^{W}\;({\hat{Y}}_{t}[c,i,j]-Y[c,i,j]{)}^{\text{2}},\; \end{array}$$ (3)

where H=W=224. We compute this loss per image and report the dataset average at each timestep; thus, the curves in Figs 1–4 are scalar MSE values per timestep, with no aggregation across timesteps. The bar plots in Figs 1–4 report the difference between MSE at t=1 (feedforward baseline) and t=15 (final predictive-coding timestep), again with no cross-timestep aggregation. All six channels contribute equally to the loss metric, without any re-weighting.

After hyperparameter optimization using a grid search strategy, both, the short and long forward models were trained using a Ranger optimizer, with a learning rate of 0.01, weight decay of 0, and a batch size of 75. The short and long backbone models were trained for 17 and 9 epochs, respectively.

The feedback models were created by adding recurrent feedback to the already trained backbone models. The activations of all encoding modules, $e_{n}$ were initiated with a feedforward pass. Then the forward weights were frozen, and the weights of the feedback transpose convolution layers, $d_{n}$, were trained using an unsupervised reconstruction objective. This seeks to minimize the reconstruction loss (prediction error), modelled as MSE between the outputs of $e_{n-\text{1}}$ and $d_{n}$.

During model inference, the predictive coding updates follow a global timestep schedule. At t=0 we run a standard feedforward pass; feedback decoders are inactive. For t=1,…,T we perform predictive-coding updates across all PCoders in parallel, where each $e_{n-\text{1}}$ updates its state using equation (2). The model output head yields a grasp map at each timestep.

All feedforward backbones were identical across model variants tested in a single experiment, and differences in trainable parameters arise only from the PCoder decoder modules (ConvTranspose2d) that implement feedback. The number of parameters in a ConvTranspose2d layer is a function of the input ($C_{\text{in}}$) and output ($C_{\text{out}}$) channel dimensions and the kernel size ($k_{h}\times k_{w}$). While kernel sizes and other hyperparameters were held constant across the *short*-, *medium*-, *long*- and *longer*-range models, $C_{\text{in}}$ and $C_{\text{out}}$ necessarily depend on the feature dimensionality at the feedback source and target layers. Consequently, the feedback model variants differ in the number of trainable parameters, reflecting their position along the backbone (i.e., level of abstraction) rather than any change to the feedforward architecture.

Hyperparameters for predictive coding dynamics, including layer-dependent balancing coefficients for the feedforward ($\beta_{n}$), feedback ($\lambda_{n}$), recurrence ($\gamma_{n}$) and error-correction ($\alpha_{n}$) terms, were optimized using a grid search strategy. The respective hyperparameters were conserved across all model variants within each experiment and are presented in Table 6.

**Table 6:** Predictive coding hyperparameters. The balancing coefficients for the feedforward ($\beta_{n}$), feedback ($\lambda_{n}$), recurrence ($\gamma_{n}$) and error-correction ($\alpha_{n}$) terms that were selected after hyperparameter optimization, are stated for the short and/or long feedforward model(s), for all experiments.

PCoder	Feedback hyperparameters	Experiments					
		1	2		3		4
		Short	Short	Long	Short	Long	Long
1	$\beta_{n}$	0.2	0.3	0.35	0.35	0.2	0.35
	$\lambda_{n}$	0.05	0.3	0.25	0.25	0.4	0.25
	$\gamma_{n}$	0.75	0.4	0.4	0.4	0.4	0.4
	$\alpha_{n}$	0.01	0.01	0.01	0.01	0.01	0.01
2	$\beta_{n}$	0.4	0.4	0.4	0.4	0.4	0.4
	$\lambda_{n}$	0.1	0	0	0	0	0
	$\gamma_{n}$	0.5	0.6	0.6	0.6	0.6	0.6
	$\alpha_{n}$	0.01	0.1	0.1	0.1	0.01	0.1
3	$\beta_{n}$	0.4	–	–	–	–	–
	$\lambda_{n}$	0.1	–	–	–	–	–
	$\gamma_{n}$	0.5	–	–	–	–	–
	$\alpha_{n}$	0.01	–	–	–	–	–
4	$\beta_{n}$	0.4	–	–	–	–	–
	$\lambda_{n}$	0	–	–	–	–	–
	$\gamma_{n}$	0.6	–	–	–	–	–
	$\alpha_{n}$	0.01	–	–	–	–	–

Model training was performed on the NVIDIA T4 and the NVIDIA QUADRO RTX 8000 GPUs.

### Dataset

Both, the backbone and feedback models, were trained on the grasp detection task using the large-scale Jacquard dataset for robotic grasp detection [29]. The Jacquard dataset is composed of 11,619 distinct objects with a total of ~5,000,000 possible grasp annotations. The grasp annotations corresponding to each image constituted a list of grasp candidate labels. Each grasp candidate was described by parameters including center pixel coordinates (x, y), orientation (theta), and dimensions (pixel width, pixel height) of the grasp. For each object instance (a distinct camera viewpoint of an object), a rendered RGB image, a segmentation mask, two depth images and the grasps annotations are available. The dataset was split into three parts: 66% for model training, 17% for model validation, and 17% for model testing. In the case of the intersection-over-union (IoU) metric calculation, only 4.5% of the dataset was used for model testing.

#### Preprocessing.

The data preprocessing pipeline transformed lists of grasp candidates into pixel-wise grasp maps. A grasp map array, of spatial dimensions corresponding to the image size (224x224), was initialized with six channels to represent the five grasp parameters and a grasp quality score map, which is a per-pixel value in [0,1] that reflects the feasibility of a grasp centered at that pixel. The preprocessing operation converted grasp annotations to bounding boxes, identified the bounding box boundaries, and iterated over each pixel within these boundaries to determine the grasp candidate closest to the pixel based on Euclidean distance. Each pixel was then labeled with the attributes of the nearest grasp and the count of valid bounding boxes covering it. The final channel (grasp quality score map), representing the count of bounding boxes per pixel, was normalized by dividing by the maximum count across all pixels. This preprocessed data format was used for training the backbone and feedback neural network models on the task of predicting grasp locations in an input image.

Feature normalization was applied to all images (RGB and depth) and grasp labels, to promote training stability and model convergence. Image pixel intensities and label values were normalized to a range between 0 and 1.

#### Additive noise.

During model inference, all test images were injected with varying levels of additive Gaussian noise of standard deviation, σ∈{0,  0.25, 0.5, 0.75, 1}.

### Calculating the IoU metric for robotic grasping

The rectangle-based IoU metric conventionally used for benchmarking robotic grasp detection [30,31,29] was additionally computed. This required decoding the model’s pixel-wise grasp-parameter maps into a set of discrete oriented grasp rectangles for comparison against the dataset’s native rectangle annotations.

For each image, the six predicted parameter channels were de-normalized to recover their physical units, inverting the normalization applied during preprocessing. Candidate grasp pixels were then identified from the confidence channel by applying spatial smoothing followed by local-maxima peak-picking; for noisy-input evaluations, the smoothing kernel was scaled with the input noise level to suppress spurious confidence peaks. Candidates were retained if their smoothed confidence exceeded a threshold (0.25), ranked by confidence, and truncated to the top-k (200) candidates. Each retained pixel was decoded into an oriented rectangle by reading the predicted offset, angle, width, and height at that pixel; non-maximum suppression (threshold = 0.25) based on oriented IoU was then applied to remove duplicate proposals.

Oriented IoU between a predicted and a ground-truth rectangle was computed by clipping their intersection polygon and dividing its area by the union area, with rectangle areas obtained via the shoelace formula. A predicted grasp was scored as a successful match to a ground-truth grasp if their oriented IoU was at least 0.25 and their absolute angular difference (modulo 180°, to account for antipodal grasp symmetry) was no more than 30°, the thresholds used in the Jacquard benchmark [29] based on conventionally used parameters. For each image, predictions were greedily matched to ground-truth grasps in descending confidence order, with each ground-truth grasp matched at most once. Image-wise success was defined as the presence of at least one matched grasp; dataset-level success was the percentage of images meeting this criterion.

### Statistics

A repeated measures analysis of variance (ANOVA) [50] was conducted to assess the effects of feedback type and noise level on the grasp detection accuracy of the model, using the Jamovi statistical software [51]. This analysis accounted for within-object effects and included tests for sphericity. Mauchly’s test of sphericity [52] was performed to check the assumption of sphericity for feedback type, noise level, and their interaction. Where the assumption was violated, Greenhouse-Geisser corrections [53] were applied to adjust the degrees of freedom. Post hoc comparisons were conducted using Tukey’s HSD test [54] to evaluate pairwise differences between the levels of feedback type.

A One-Way ANOVA [55] was conducted to compare the difference in model performance between the first and final timestep during the test, across different noise levels [51].

**Significance statement**

It is commonly held that the brain predicts the causes of its sensorium via top-down neural pathways. While canonical models of predictive coding assume reciprocal feedforward and feedback connections, functional evidence highlights the importance of non-reciprocal ‘asymmetric’ feedback, whose role remains poorly understood. Using neural network models of grasp prediction, we characterized optimal pathlengths and source regions for asymmetric feedback facilitating visual processing of noisy sensory inputs. Our findings show that *medium*-range feedback from early layers marks a sweet spot, incorporating optimal distance between the neural representations of source/target layers and representational abstraction of the feedback source. This intimates an uncharted role of intermediate brain areas along the visuomotor stream as a source of predictive signals.

## Supporting information

S1 File Copyright permission. (PDF)
