# Supplementary material for: Predictive coding explains asymmetric connectivity in the brain: A neural network study
Source: PLoS Comput Biol. 2026 Jul 6;22(7):e1014435. doi: 10.1371/journal.pcbi.1014435 (PMC13336203; doi:10.1371/journal.pcbi.1014435)
Supplement: S1 File — (PDF) [file pcbi.1014435.s001.pdf]

Outlook

Re: Permission request: using Jacquard dataset images in a publication

From Emmanuel Dellandrea <emmanuel.dellandrea@ec-lyon.fr>
Date Wed 5/20/2026 2:41 AM
To Romesa Khan <romesa.khan@mail.utoronto.ca>

You don't often get email from emmanuel.dellandrea@ec-lyon.fr. Learn why this is important

Dear Romesa,
You can use the Jacquard dataset images in you manuscript under this license.

Best regards.

--
Emmanuel Dellandréa
Maître de Conférences HDR / Associate Professor
Centrale Lyon - Laboratoire LIRIS (UMR 5205)
http://perso.ec-lyon.fr/emmanuel.dellandrea/

De: "Romesa Khan" <romesa.khan@mail.utoronto.ca>
À: "jacquard" <jacquard@liris.cnrs.fr>
Envoyé: Mardi 5 Mai 2026 22:24:01
Objet: Permission request: using Jacquard dataset images in a publication

Dear Dr. Depierre, Dr. Dellandréa, and Dr. Chen,

I am writing to request permission to reproduce a small number of object images from the Jacquard dataset (Depierre, Dellandréa, & Chen, 2018) in a manuscript currently under revision at *PLOS Computational Biology*.

The manuscript uses the Jacquard dataset to train and evaluate convolutional neural network models. The dataset is fully cited in the Methods, References, and figure captions.

PLOS Computational Biology publishes all content under the Creative Commons Attribution 4.0 International (CC BY 4.0) license, which permits reuse with attribution. To comply with this requirement, the journal asks that I obtain written permission from the copyright holder authorizing publication under CC BY 4.0.

Could you kindly confirm by reply whether you grant permission for the inclusion of these Jacquard dataset images in the manuscript under the CC BY 4.0 license?

I would be grateful for your response at your earliest convenience, as the manuscript is currently in active revision. Please let me know if any additional information would be helpful.

Thank you very much for your time and for making the Jacquard dataset available to the research community.

Best regards,

Romesa Khan

Department of Psychology

University of Toronto Scarborough
